# Allelic RNA Motifs in Regulating Systemic Trafficking of Potato Spindle Tuber Viroid

**DOI:** 10.3390/v10040160

**Published:** 2018-03-30

**Authors:** Ryuta Takeda, Craig L. Zirbel, Neocles B. Leontis, Ying Wang, Biao Ding

**Affiliations:** 1 Molecular, Cellular, and Developmental Biology Program, Ohio State University, Columbus, OH 43210, USA; 2 Department of Mathematics and Statistics, Bowling Green State University, Bowling Green, OH 43403, USA; zirbel@bgsu.edu; 3 Department of Chemistry and Center for Biomolecular Sciences, Bowling Green State University, Bowling Green, OH 43403, USA; leontis@bgsu.edu; 4 Department of Molecular Genetics, Ohio State University, Columbus, OH 43210, USA; 5 Department of Biological Sciences, Mississippi State University, Starkville, MS 39762, USA

**Keywords:** viroid, RNA trafficking, RNA structural motifs

## Abstract

Intercellular RNA trafficking has been shown as a widely-existing phenomenon that has significant functions in many aspects of biology. Viroids, circular noncoding RNAs that cause plant diseases, have been a model to dissect the role of RNA structural motifs in regulating intercellular RNA trafficking in plants. Recent studies on potato spindle tuber viroid (PSTVd) showed that the RNA motif loop 19 is important for PSTVd to spread from palisade to spongy mesophyll in infected leaves. Here, we performed saturated mutational analysis to uncover all possible functional variants of loop 19 and exploit this data to pinpoint to a three-dimensional structural model of this motif. Interestingly, we found that two distinct structural motifs can replace loop 19 and retain the systemic trafficking capacity. One of the alternative structures rapidly emerged from the inoculation using a loop 19 abolished mutant that is not capable of systemic trafficking. Our observation indicates the flexibility of multiple structural arrangements interchangeably exerting similar function at a particular RNA locus. Taken together, this study deepens the understanding of RNA structural motifs-regulated viroid RNA trafficking, which has broad implications for studying RNA intercellular trafficking as well.

## 1. Introduction

Multicellular organisms rely on various signals to integrate their growth and responses to environmental cues. RNA intercellular trafficking, including short distance cell-to-cell and long distance systemic movement, has been demonstrated as a critical means for cellular communications in various organisms [1,2,3,4]. Viruses and sub-viral agents also exploit such pathways to successfully invade a host [5,6,7,8]. Being a special group of sub-viruses and made up of only circular noncoding RNAs, viroids entirely depend on their RNA structural motifs to interact with host factors for function [7,9,10]. The RNA genome of potato spindle tuber viroid (PSTVd, species *Potato spindle tuber viroid*, family *Pospiviroidae*) is composed of 359 nt folding in a rod-like secondary structure comprising 27 RNA loop motifs flanked by short double helices (Figure S1). PSTVd loop motifs are designated numerically in order from the left terminal loop (loop 1) to the right terminal loop (loop 27) (Figure S1). Base changes that replace these loop sequences with Watson-Crick (WC) base pairings (the “loop-closing” experimental strategy) unraveled 11 out of 27 loop motifs critical for systemic trafficking of PSTVd, attesting to the functional relevance of those RNA motifs [11]. 

Most of these 27 RNA loop motifs form RNA 3D motifs via non-WC base-pairings and other base-specific interactions, including stacking and base-backbone interactions, supported by extensive chemical mapping assays [12,13,14]. In an RNA structural motif, each RNA base utilizes its three edges (i.e., Watson Crick (WC), Hoogsteen, and sugar edge) for possible base-pairings. Considering the relative positions of glycosidic bonds, there are “*cis*” or “*trans*” orientations. Taken together, there are 12 base-pairing geometries [15]. Base-pairings occupying similar space, termed isosteric base-pairings, are likely interchangeable without disrupting the 3D structure or the related function. These isosteric base-pairings should use the same edges for interactions, share the same orientations, and occupy the same C1′-C1′ distance in space [16]. Considering their biological importance, RNA structural motifs act as a critical factor constraining the evolution of viroids and, potentially, other RNA viruses [17]. This constraint operates because mutations in a 3D motif maintaining the structure and function will be retained in the population whereas mutations disrupting the 3D structure and, consequently, the function, will be lost [17]. Recent studies have shown that RNA structural motifs control the possible PSTVd sequence variations that are capable of systemic spreading in plants [18,19,20,21]. Taking PSTVd loop 6 as an example, this 3X3 loop has 4^6^ possible sequence combinations but only eight isosteric variants are functional among 49 possible isosteric base-pairings (including wild-type) [22]. It is apparent that isosteric base-pairings constrain the possible sequence variations by >80 folds (4^6^/49).

Here we focused on loop 19 (Figure S1), which resides in the variable domain [23] and is known as a functional loop for the long-distance spreading of PSTVd in *Nicotiana benthamiana* [11,24]. When introducing WC base pairing to replace the non-WC “A●C” motif, the mutant retained the capacity to replicate, but failed to traffic to systemic leaves in twelve test plants [11]. A recent report showed that this loop may be required for PSTVd to enter spongy mesophyll from palisade mesophyll in inoculated leaves [24]. We performed saturated mutagenesis and revealed all possible sequence variations retaining the systemic trafficking and replication capacity. These data laid a foundation to pinpoint to a structural model annotating the non-WC base-pairing pattern therein. Interestingly, we also identified alternative structural arrangements interchangeable at the same locus to retain the PSTVd trafficking capacity, expanding the current understanding regarding how structural motifs control the viroid systemic trafficking while constraining the viroid evolution. This knowledge has implications in understanding intercellular trafficking of viral and host RNAs.

## 2. Materials and Methods

### 2.1. Plant Materials and Growth Conditions

All plants were grown in a growth chamber controlled at the 14-h light, 27 °C/10-h dark, 24 °C cycles. PSTVd infection was achieved by inoculation 200 ng of (+)-PSTVd *in vitro* transcripts onto four-true leaf-stage seedlings of *N. benthamiana*. PSTVd infection (28 days post-inoculation) was verified by Northern blotting. 

### 2.2. RNA Extraction and Northern Blotting

Total RNA from mock- or PSTVd-infected leaves was purified using Trizol reagent (Thermo Fisher Scientific, Waltham, MA, USA) following manufacturer’s instructions. Northern blotting was previously described in detail [25].

### 2.3. Molecular Cloning

To examine the sequence identity of progeny, reverse transcription was performed using SuperScript III transcriptase (Thermo Fisher Scientific) and PSTVd specific reverse primer (PSTVd r: 5′-AGGAACCAACTGCGGTTCCA-3′), followed by regular PCR using Pfu Turbo DNA polymerase (Thermo Fisher Scientific) and primers (PSTVd f: 5′-CGGAACTAAACTCGTGGTTCCT-3′; PSTVd r: 5′-AGGAACCAACTGCGGTTCCA-3′). The PCR product was treated with Taq polymerase (Genscript, Piscataway, NJ, USA) to add an A-tail and followed by ligation to the pGEM-T vector (Promega, Madison, WI, USA). The positive clones were subject to Sanger sequencing. The plasmid templates for in vitro transcription were generated via site-directed mutagenesis. We used pRZ6-2 harboring PSTVd^Int^ [26] as a template following protocols described in details previously [25]. Template (pInter-) for generating the Northern blotting probe was described before [27]. All constructs were verified by DNA sequencing at the OSU Plant-Microbe Genomics Facility.

### 2.4. In Vitro Transcription

To prepare RNA inoculum from wild-type (WT) and PSTVd variants, HindIII-linearized pRZ6-2 plasmids harboring correct PSTVd sequences served as templates for T7 polymerase transcription (Thermo Fisher Scientific) following manufacturer’s instructions. The generated RNA was purified using a MEGAClear kit (Thermo Fisher Scientific) after DNase I treatment. SpeI-linearized pInter-template was subject to in vitro transcription using a T7 MAXIscript kit (Thermo Fisher Scientific). The product, [α-^32^P] UTP-labeled riboprobes, was used for Northern blotting.

## 3. Results 

### 3.1. Discovering Functional Loop 19 Variants through Saturated Mutational Analysis

**Figure 1:**
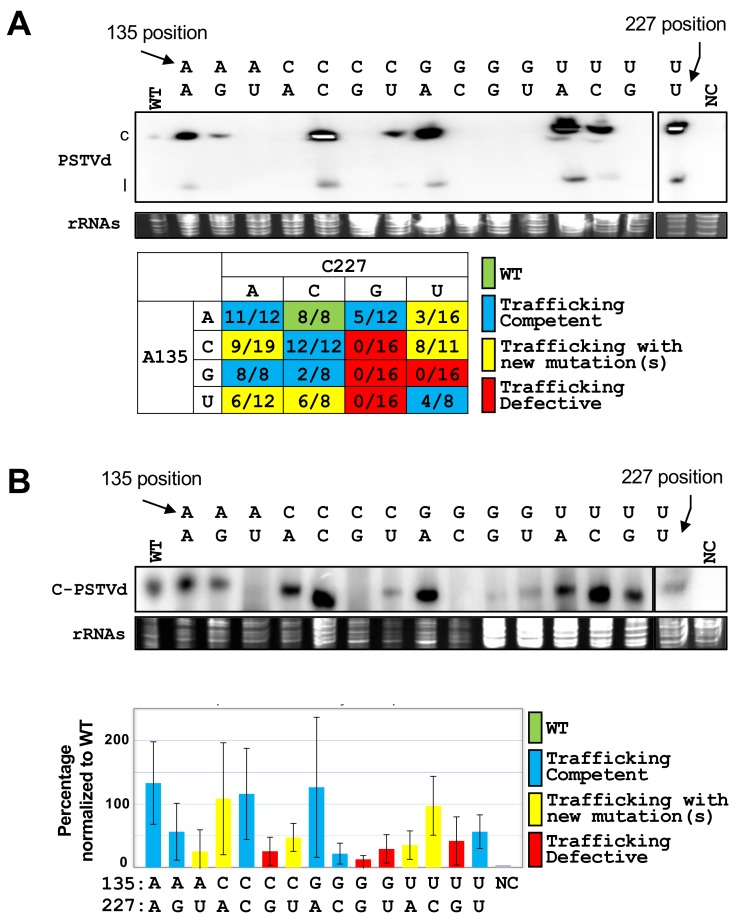
Saturated mutational analyses revealing the functional loop 19 variants of PSTVd. (**A**) Northern blots showed the accumulation of functional loop 19 variants accumulated in systemic leaves in one infection. Each variant was inoculated with at least eight biological replicates, and the data were summarized in the table. c, circular PSTVd. l, linear PSTVd; (**B**) One of the triplicated Northern blots showed the accumulation of progeny from the replication of loop 19 variants in inoculated leaves. The replication efficiency of all variants was normalized to that of WT and is shown in the lower panel. rRNAs served as the loading control. C-PSTVd, circular PSTVd. WT, wild-type. NC, negative controls.

Loop19 is known as a critical loop for the intercellular trafficking of PSTVd^Int^ in *N. benthamiana* [11], likely regulating its entry into spongy mesophyll from palisade mesophyll [24]. Mutational analysis showed that replacing the loop with a canonical WC base-pairing abolished the function, indicating the existence of some non-canonical base-pairing for function [11]. To better understand the possible variants possessing the trafficking capacity, we generated all 16 possible loop 19 variants (including WT) and tested their systemic infection capacity. In summary, four variants (“C●G”, “G●G”, “G●U”, and “U●G”) showed completely no systemic trafficking, five variants (“A●U”, “C●A”, “C●U”, “U●A”, and “U●C”) occasionally trafficked to systemic leaves in a few tested plants, but with additional mutations that complicated the data annotation, two variants (“A●G” and “G●C”) showed limited trafficking capacity with a lower-than-50% success rate, while the rest five variants (including the WT “A●C” pair) showed optimal rates for systemic trafficking. An example of Northern blotting results and the summary of trafficking results are shown in Figure 1A. Our result is in line with the recent report where the same four variants (“C●G”, “G●G”, “G●U”, and “U●G”) showed no systemic trafficking capacity [24]. We also examined the accumulation of the circular form RNA of these variants in inoculated leaves, with three biological replicates. As representative data show in Figure 1B, mutants with “A●U” and “G●C” pairings showed almost neglected accumulation of circular PSTVd in inoculated local leaves, indicating the functional deficiency of both variants. It is noteworthy that the deficiency of the “G●C” variant in accumulation in inoculated leaves explains its low rate of systemic trafficking. The mutant with the “C●G” pairing showed reasonable replication in one replicate. The replication efficiency of all variants was normalized to that of WT based on triplicates and listed in Figure 1B.

### 3.2. The Possible Structural Model for PSTVd Loop 19

**Figure 2:**
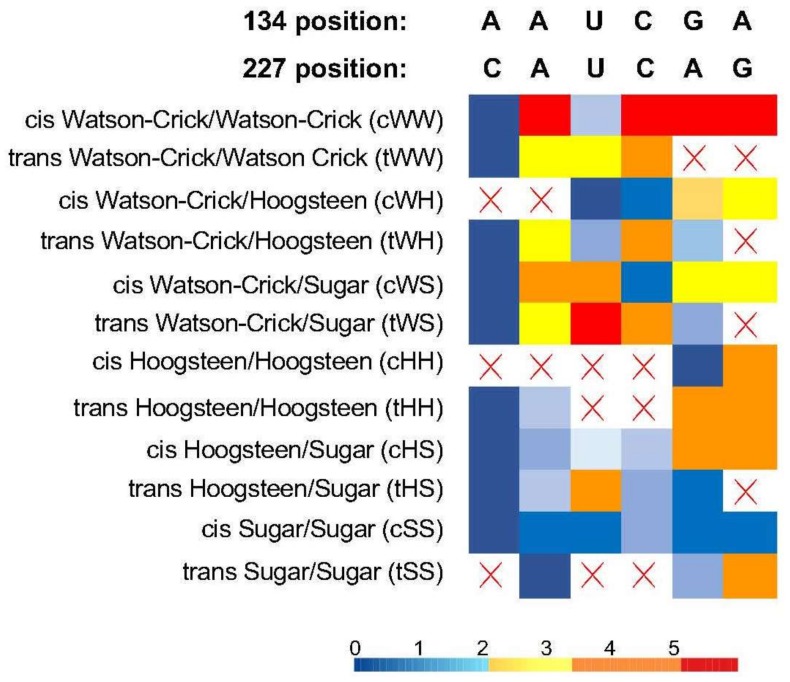
The heat map view of structural similarity among six function loop 19 variants. Red crosses dictate that the structures cannot form using this base pairing geometry. The numeric value in the IsoDiscrepancy Index heat map: values less than 2.2 are in blue color depicting isosteric base pairings; values between 2.2 and 3.5 are displayed in yellow color depicting nearly isosteric base pairings; values above 3.5 are colored in orange or red depicting the non-isosteric base pairings.

Previous studies utilized the homology-based algorism to predict the 3D structures of PSTVd motifs [22,28,29]. However, it is not applicable for loop 19 structural analyses because using this simple loop as a bait for sequence homology-based search retrieved too many possible structures with similar likelihood scores when using the latest program JAR3D [30]. To circumvent this difficulty, we relied on the sequences of the six functional variants and assumed that these functional variants are isosteric. This assumption was based on the latest knowledge that RNA 3D structural motifs constrain viroid evolution [17], as well as multiple previous observations that functional variants of a given RNA motif are all isosteric [22,25,28]. Since there are 12 possible base-pairing geometries for loop 19, we analyzed the structural similarities among the six fully functional variants when they were placed in any given one of the 12 possible geometries (Figure 2), using RNA Basepair Catalog (http://ndbserver.rutgers.edu/ndbmodule/services/BPCatalog/bpCatalog.html). The RNA Basepair Catalog utilized a heat map (with scores) to show the structural similarities among all possible base pairings within a given geometric group. The darker the blue color, the more similar in 3D structures (isosteric) the two base-pairings are. Whereas the darker the red color, the more dissimilar 3D structures (non-isosteric) the two base-pairings are. In practice, we first assumed that the base-pairing in loop 19 forms one of 12 geometry, here taking *cis* Watson-Crick/Watson-Crick base-pairing pattern as an example, then we checked the structural similarities among the six functional variants when they form *cis* Watson-Crick/Watson-Crick base-pairing. In this case, we found that the WT “A●C” is only weakly similar to “U●U” (light blue) while there are four functional variants not isosteric to WT (dark red) (Figure 2). After listing the geometric similarities among all the six functional variants in all 12 possible geometries, we found that these variants are isosteric to each other only if they form the *cis* sugar/sugar base-pairing pattern (Figure 2). Therefore, we concluded that two nucleotides probably interact via forming a *cis* sugar/sugar base-pairing in PSTVd loop19.

### 3.3. De Novo Emergence of a Loop 19-Like Structure When Inoculating with PSTVd^IntC227U^

**Figure 3:**
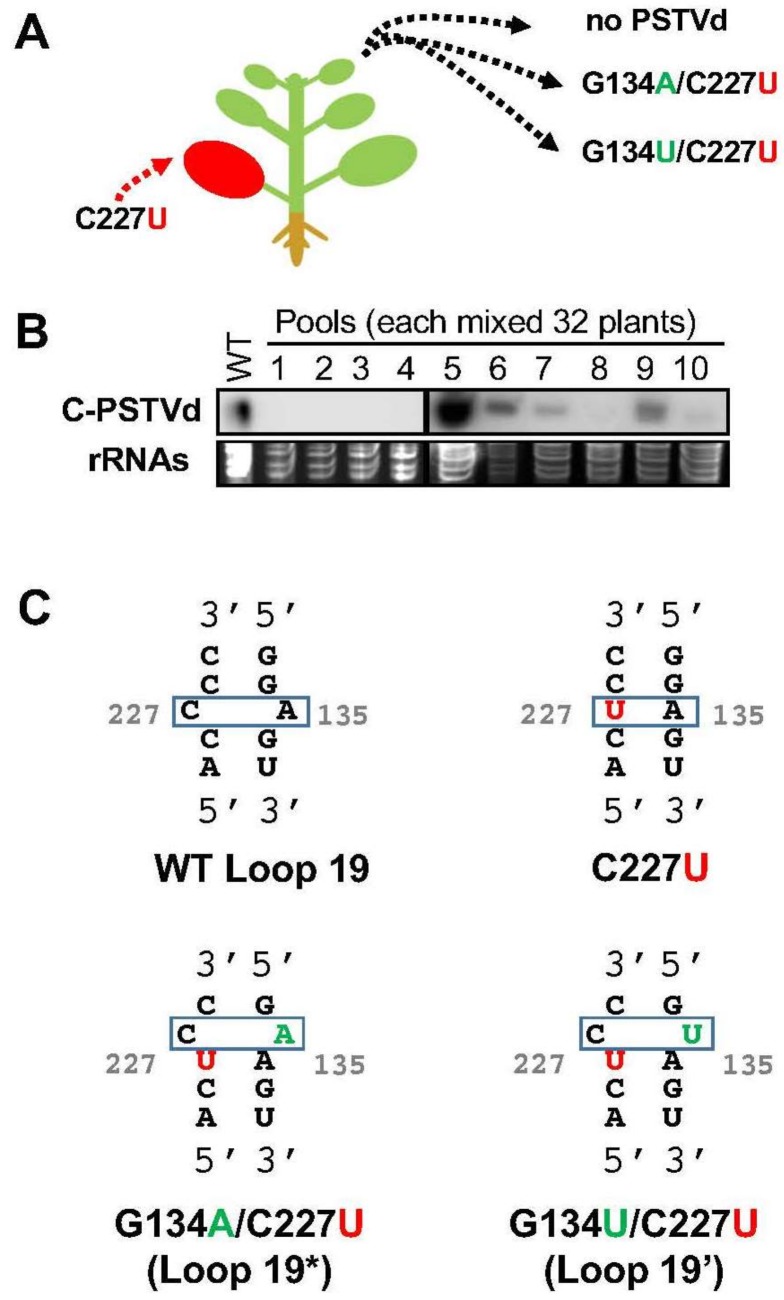
Rapid emergence of functional RNA motifs. (**A**) Inoculation of PSTVd^IntC227U^ mutant led to the emergence of new variants in progeny, which showed up in systemic leaves; (**B**) Northern blots showed the presence of PSTVd in pooled samples from systemic leaves. C-PSTVd, circular PSTVd. rRNAs served as the loading control; and (**C**) illustrations of loop 19* and loop 19′. The U at the 227 position in the mutant background is highlighted in red, and the newly-emerged substitutions of A or U at 134 position are highlighted in green.

Interestingly, we also noticed the emergence of new variants when inoculated with the PSTVd^IntC227U^ mutant, which is trafficking-deficient. We infected 320 plants with PSTVd^IntC227U^ RNA and examined infection 28 days post inoculation. The 320 plants were divided into 10 groups with each group containing 32 plants. To check infection, we pooled leaves from all 32 plants in each group as one sample and found the presence of PSTVd in systemic leaves indicated by strong signals of four pools and a weak signal from one pool, based on Northern blotting (Figure 3). To elucidate the characteristics of the progeny population that traffic systemically, RNA was extracted from the systemic leaves of seven randomly-chosen plants with infection signal and cloned PSTVd from systemic leaves via RT-PCR and sequenced (Table S1). It is noteworthy that all the sequenced progeny acquired a *de novo* mutation at the G134 position while the C227U was maintained. Five of the sequenced progeny acquired a G134A substitution and the other two had the G134U substitution (Figure 3C). Additional substitutions were observed in regions other than loop 19 (U27C, G67C, U200A, U200C, and G319U) in four of the sequenced progeny, but they were predicted to have no impact on local structure in the loop 19 region based on mFOLD [31].

We found the PSTVd^IntG134A/C227U^ variant particularly interesting because of its high frequency observed among the progeny. The mFOLD [31] program predicted that G134A substitution in PSTVd^IntC227U^ restored the loop 19 “A●C” single non-WC base-pairing next to the position where the original loop 19 resides in PSTVd^Int^. We designated this new strain, PSTVd^IntG134A/C227U^, as loop 19*. Interestingly, G134U displayed a similar pattern that generated the non-WC base pair “U●C” (PSTVd^IntG134U/C227U^), which we designated as loop 19′.

**Figure 4:**
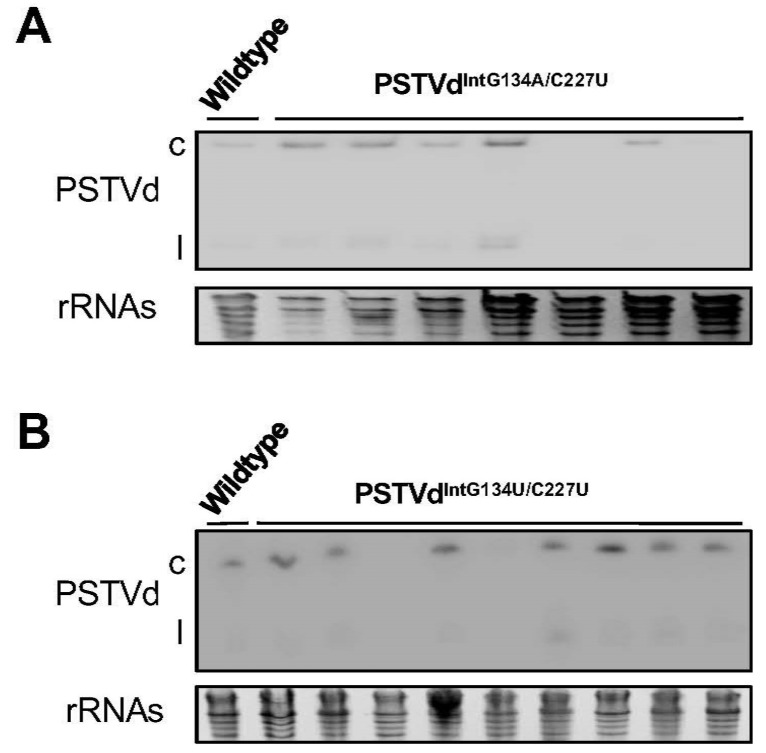
Infection assay showing the trafficking capacity of loop 19* and loop 19′. (**A**) Northern blots showed the accumulation of loop 19* (PSTVd^IntG134A/C227U^) in systemic leaves; (**B**) Northern blots showed the accumulation of loop 19′ (PSTVd^IntG134A/C227U^) in systemic leaves. rRNAs served as the loading control. c, circular PSTVd. l, linear PSTVd.

To be present in systemic leaves, PSTVd^IntG134A/C227U^ and PSTVd^IntG134U/C227U^ may be functional variants or generated after their emergence through replication errors from other variants. To sort out the possibilities, we further checked the systemic trafficking function of each of these mutants with the infection assay. We inoculated plants with PSTVd^IntG134A/C227U^ or PSTVd^IntG134U/C227U^ transcripts and detected their trafficking to systemic leaves 28 days post inoculation using Northern blotting. Both variants successfully trafficked to systemic leaves in most of the test plants (five out of seven plants and seven out of nine plants, respectively), comparable to WT (Figure 4). We then sequenced the progeny obtained from systemic leaves using RT-PCR and cloning. The progeny sequences (Table S1) showed that the inoculum sequences were maintained in at least one test plant, thus supporting the functionality of loop 19* and loop 19′. Thus, we conclude that both variants are *bona fide* “gain-of-function” variants resembling WT PSTVd. 

### 3.4. A Larger (2X2) Motif Can Replace Loop 19 and Retain the Capacity for Systemic Trafficking

**Figure 5:**
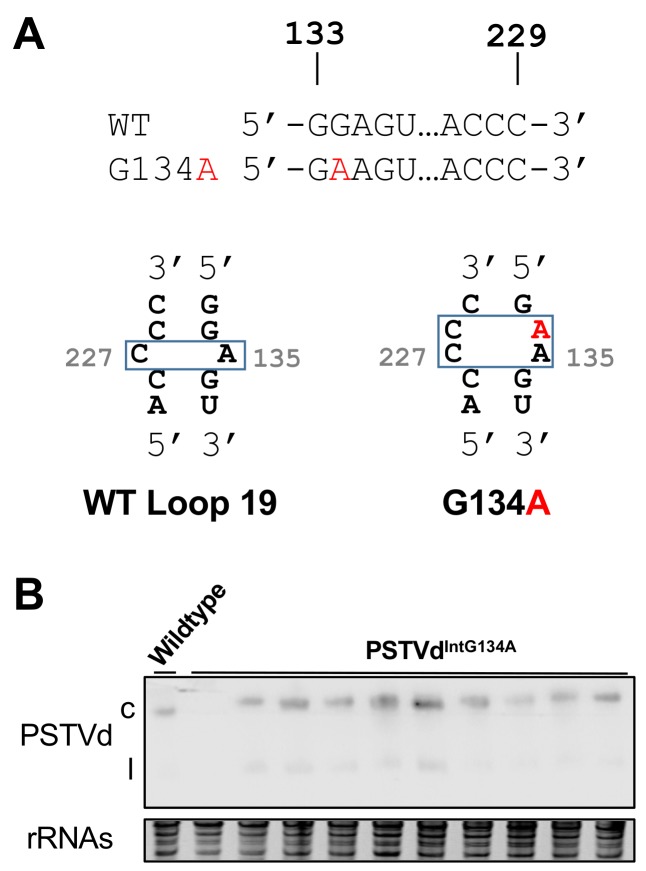
Systemic trafficking analyses on the G134A variant. (**A**) The illustration of WT loop 19 and the corresponding structure of PSTVd^IntG134A^. The primary sequence alignment is listed on top, with the G134A change highlighted in red; and (**B**) the trafficking capacity of PSTVd^IntG134A^ was demonstrated by their presence in systemic leaves, using Northern blots. rRNAs served as the loading control. c, circular PSTVd. l, linear PSTVd.

In 156 registered PSTVd sequences from the sub-viral RNA database [32], we found 152 of them sharing the same loop 19 sequence and presumably also possess the same local RNA structure by performing sequence alignment analyses using ClsustalW (Version 2.0; http://www.genome.jp/tools-bin/clustalw) [33]. The exceptions are AF483470, AY372398, EF459701, and X17268 (these IDs can be used to retrieve full-length sequences in NCBI nucleotide database). X17268 is a C227U variant that has been shown to be incapable in systemic trafficking [11]. However, the remaining three have the G134A substitution that gives rise to a larger loop (Figure 5A). Therefore, it is possible that the deeply-conserved loop 19 sequence is critical for PSTVd systemic trafficking in a host plant, but other structural arrangements may exist to exert similar functions.

We generated this variant PSTVd^IntG134A^ using site-directed mutagenesis and tested its trafficking. We observed trafficking in nine out of ten plants (Figure 5B) while the progeny sequence was maintained in at least two out of three test plants (Table S1). This supports the infectivity of PSTVd^IntG134A^. 

## 4. Discussion

Loop 19 is known as a critical loop for systemic spreading of PSTVd in *N. benthamiana* [11]. When introducing a WC base-pairing to replace the non-WC “A●C” motif, the mutant retained the capacity to replicate, but failed to traffic to systemic leaves in twelve test plants [11]. A recent report showed that this loop may be important for PSTVd to enter spongy mesophyll from palisade mesophyll in inoculated leaves [24], which resembles the function of a previously reported RNA 3D motif, PSTVd loop 6 [22]. Two distinct motifs critical for the same trafficking route of a given RNA supports a complicated, but coordinated, mode of multiple structures in regulating the cell-to-cell movement of RNAs [34]. Despite its critical function in regulating PSTVd systemic trafficking, less is known regarding how the two nucleotides interact in 3D space to lend a structural basis for this function. Through the saturated mutational analysis, we first identified all six functional variants. We then searched for a base-pairing geometry in which all the functional variants are isosteric, which is a critical constraint for the presence of possible functional variants. As a result, our analysis pinpoints to only one possible geometry, the *cis* sugar/sugar base-pairing, for PSTVd loop 19.

Viroids have high mutation rates in general. The chloroplastic viroids have the highest mutation rates in all living entities [35] and the nuclear viroids have relatively lower mutation rates resembling some RNA viruses [36]. When introducing designed PSTVd mutants for infection, reversion to WT or other functional sequences has been frequently observed [37]. For example, five nucleotide-substitutions, forming a so-called bipartite motif, converted PSTVd^NT^ to PSTVd^NB^, the latter of which is capable of moving from the bundle sheath to the mesophyll in tobacco leaves [38]. However, the structural arrangements of this bipartite motif remain poorly understood. When infecting using PSTVd^KF-440/U187A^, reversion to WT occurred to restore systemic infection in tomato plants. This single nucleotide change restored a partially disrupted structure to function [37]. Interestingly, several point mutations in the loop E motif enabled PSTVd to embark on infection in tobacco [39,40], which showed that subtle changes in primary sequences can potentiate new functions while maintaining the isosteric structures of an RNA motif [17,25]. 

Here, we provided evidence for the rapid *de novo* emergence of a functional RNA structural motif within a helix region in regulating PSTVd systemic trafficking. As long as the newly-emerged structure is compatible with the selection pressure, it can be kept in the quasispecies and allows a new function of a pathogen. This finding supports that viroids can evolve a completely new RNA structural motif to help establish novel tissue tropisms. More interestingly, due to fewer constraints, several distinct structures may emerge at the same locus in different individuals to perform similar functions. It is generally assumed that viroids rely entirely on their structural motifs to interact with host factors for function. Distinct structural motifs, being interchangeable at the same locus to exert similar function, reflect the flexibility of host factors in recognizing functional structural motifs, highlighting the complexity of host machinery for RNA systemic trafficking. It is also notable that this *de novo* emergence of a novel RNA structural motif can occur at a relatively fast rate (less than 28 days in this report), which may help better understand the molecular basis underlying host-viroid/virus interactions.

## Supplementary Materials

The following are available online at http://www.mdpi.com/1999-4915/10/4/160/s1, Figure S1. PSTVd secondary structure and loop 19. The secondary structure of PSTVd^Int^ genome is shown, with all the loops labeled numerically in blue. The five known domains are highlighted by dashed grey lines. The genomic locus of loop 19 is highlighted in a red box. The deeply-conserved loop 19 structure and a naturally-existing variant are shown in the lower panel. Table S1. Sequences of progeny from systemic leaves of infected plants.
