# Supplementary material for: Allelic RNA Motifs in Regulating Systemic Trafficking of Potato Spindle Tuber Viroid
Source: Viruses. 2018 Mar 30;10(4):160. doi: 10.3390/v10040160 (PMC5923454; doi:10.3390/v10040160)
Supplement: Supplementary file 1 [file viruses-10-00160-s001.zip › Supplementary files/2018-3-28 Figure S1.pptx]

## Slide 1
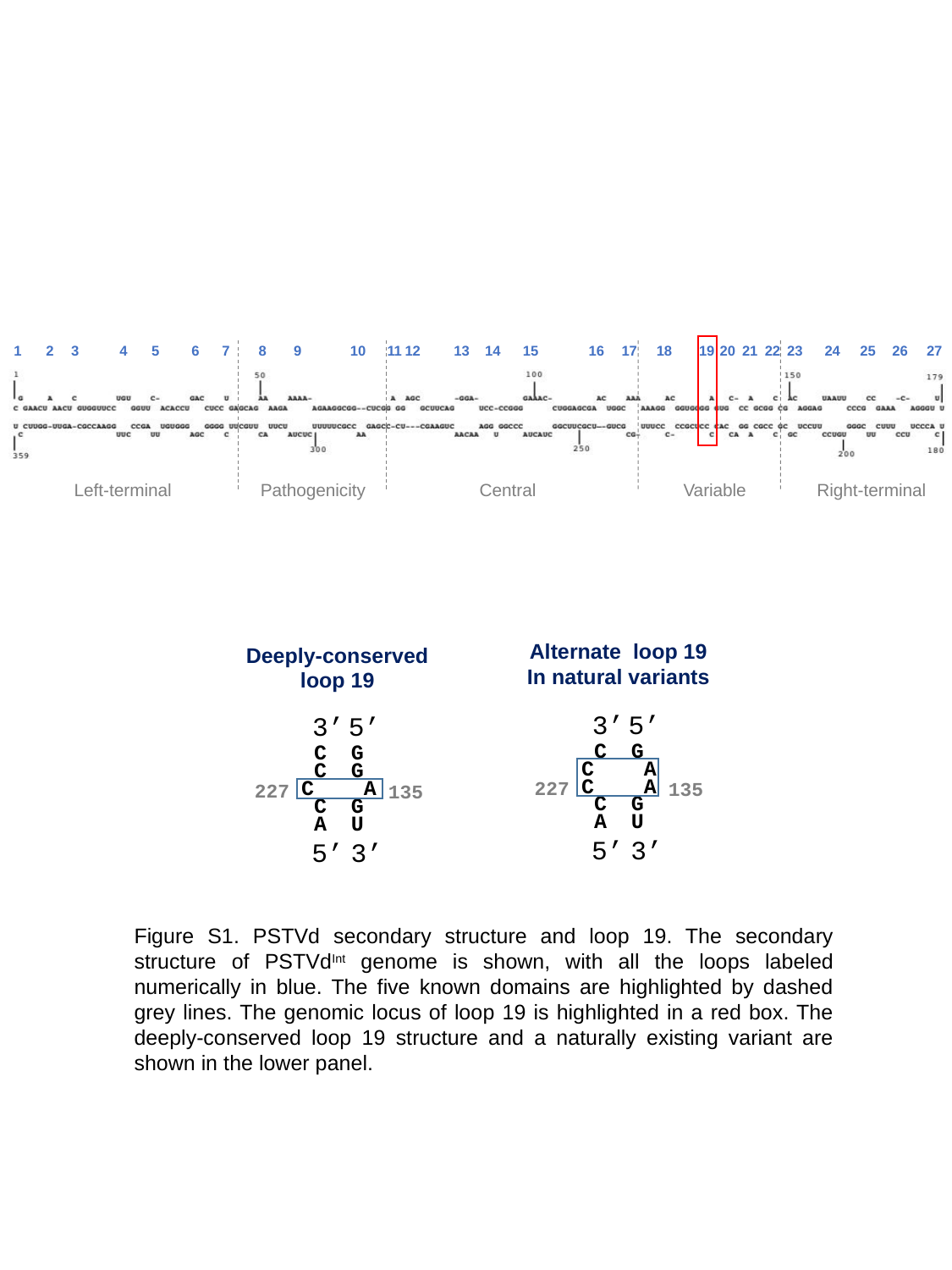

1
2
3
4
5
6
7
8
9
10
11
12
13
14
15
16
17
18
19
20
21
22
23
24
25
26
27
Left-terminal
Pathogenicity
Central
Variable
Right-terminal
Alternate loop 19
In natural variants
Deeply-conserved loop 19
3’
5’
 C
C
C
 C
 A
G
 A
 A
G
U
227
135
5’
3’
3’
5’
 C
 C
C
 C
 A
G
G
 A
G
U
227
135
5’
3’
Figure S1. PSTVd secondary structure and loop 19. The secondary structure of PSTVdInt genome is shown, with all the loops labeled numerically in blue. The five known domains are highlighted by dashed grey lines. The genomic locus of loop 19 is highlighted in a red box. The deeply-conserved loop 19 structure and a naturally existing variant are shown in the lower panel.
